# The impact of moulage on learners’ experience in simulation-based education and training: systematic review

**DOI:** 10.1186/s12909-023-04976-w

**Published:** 2024-01-03

**Authors:** Stacia DCosta, Grace Zadow, Dianne P. Reidlinger, Gregory R. Cox, Carly Hudson, Ale Ingabire, Jessica Stokes-Parish

**Affiliations:** Faculty of Health Sciences and Medicine, Bond University, 14 University Drive, Robina, 4226 Australia

**Keywords:** Assessment, Education, Learner experience, Moulage, Simulation-based education training, Health professions education

## Abstract

**Background:**

Moulage is a technique used to simulate injury, disease, aging and other physical characteristics specific to a scenario, often used in health and emergency worker training, predominantly for simulation-based learning activities. Its use in allied health fields is unclear. Previous work has explored moulage as an adjunct for authentic simulations, however there is opportunity for broadening its scope.

**Aim:**

To explore the effects of moulage interventions in simulation-based education and training, for learner experience. A secondary aim was to understand which pedagogical frameworks were embedded in moulage interventions.

**Method:**

Four electronic databases (PubMed, CINAHL, EmBase, Proquest Central) were systematically searched to December 2022 for studies utilising moulage in simulation-based education experiences. Outcomes were focused on learner satisfaction, confidence, immersion, engagement, performance, or knowledge. Study quality was assessed using the Mixed Methods Appraisal Tool.

**Results:**

Twenty studies (*n* = 11,470) were included. Studies were primarily conducted in medicine (*n* = 9 studies) and nursing (*n* = 5 studies) and less frequently across other health disciplines. The findings demonstrated greater learner satisfaction, confidence, and immersion when moulage was used against a comparator group. Minimal improvements in knowledge and performance were identified. One study underpinned the intervention with a pedagogical theory.

**Conclusion:**

Moulage improves learner experience in simulation-based education or training, but not knowledge or clinical performance. Further research utilising moulage across a broader range of professions is needed. Interventions using moulage should be underpinned by pedagogical theories.

**Supplementary Information:**

The online version contains supplementary material available at 10.1186/s12909-023-04976-w.

## Introduction

Simulation-based education (SBE) and training allows learners to practice skills, decision-making, and critical thinking in a safe, supervised environment [1]. Thus, it improves preparedness for placement and develops empathetic behaviours [2]. It aims to closely mimic real-life scenarios and offer learners the opportunity to refine skills, particularly those required for less common but crucial situations [3, 4]. The use of simulation-based education and training has been well-documented across nursing and medical curricula [1] and has been adopted in allied health professions such as speech pathology, physiotherapy, audiology, and dietetics [5–7].

The reported effectiveness of simulation-based education and training for learning can be understood through its basis in multiple learning theories and frameworks [2]. For example, consider Kolb’s Experiential Learning Cycle (Kolb, 1984). It encompasses four stages of the learning process: concrete experience, reflective observation, abstract conceptualization, and active experimentation. SBE and training can provide learners with all four stages, offering hands-on learning in safe environments, with or without peer observation, to deliver concrete experiences and experimentation in simulated sessions with guided reflection [8].

A feature increasingly utilised in simulation experience, known as moulage, attempts to increase the realism of cases used in learning activities through enhancing sensory properties, thus contributing to a believable simulation experience. Modern moulage techniques include special effects makeup techniques on simulated participants or manikins. Methods can include applying castings and moulded wounds; painting bruises, lacerations or rashes; creating an illusion of blood loss, or signs of illnesses on the skin, or any other clinical presentation [9]. Moulage has been used extensively within the field of dermatology to provide an opportunity to educate individuals in various skin ailments including melanoma [10].

Several theories inform moulage use in simulation, including the theories of realism, authenticity, and fidelity [11–13]. Realism describes how a participant perceives the reality of a simulated environment, while authenticity describes how close to reality something is [14]. For this paper, we will discuss moulage in the context of realism and authenticity, but not fidelity, due to the contentious nature of the term fidelity [15–17]. Moulage use is informed by Dieckmann’s theory of realism (2007) in which realism comprises three elements: physical, semantic, and phenomenal [11]. These three aspects contribute to how a participant might perceive reality. This is particularly important when it comes to moulage, as moulage can contribute to all three aspects of realism [14]. That is, moulage provides physical prompts, conceptual prompts (e.g., bleeding means low blood pressure), and semantic prompts (e.g., moulage contributes to emotional engagement). The theory of authentic learning argues four key aspects are essential: “real worldness”, open ended inquiry, discourse among learners, and choice [12]. Moulage can contribute to authentic learning experiences by providing “real worldness” that narrows the gap between real and simulated worlds.

The Society of Simulation in Healthcare, defines moulage as:

*“A technique used to simulate injury, disease, aging and other physical characteristics specific to a scenario; moulage supports the sensory perceptions of participants and authenticity of the simulation scenario through the use of makeup, attachable artefacts (e.g. penetrating objects), and smells.”* [18].

Moulage use is emerging in paramedicine, radiography, medical education, rescue ambulance services, however largely exists in discipline silos [19]. Military simulations utilise moulage techniques to aid in desensitisation, prepare for battle and provide opportunities to practice injury treatment [20]. Recent advancements in moulage include the use of temporary tattoos to represent injuries during virtual reality training to increase the preparedness of healthcare students responding to mass casualty incidents [21].

Moulage has been shown to assist in content and face validity of simulated learning experiences, along with the transfer of clinical skills and knowledge retention [22]. Comparative studies of moulage versus no moulage have shown that moulage demonstrated improvement in learners’ performance and immersion in their clinical scenario [23]. A previous systematic review exploring the role of moulage in simulation practice and the degree to which its authenticity impacts engagement, identified that further research into the use of moulage was warranted [24]. What is yet to be understood is how moulage contributes to the learner’s experience and the role that moulage plays in educational outcomes. Therefore, this systematic review aims to explore the effect of moulage in simulation-based education and training on learner experience. A secondary aim is to describe the pedagogical frameworks and/or learning theories that can aid in better simulation experiences.

## Methods

### Search strategy

The search strategy was developed using the PICO tool (population, intervention/exposure, comparison and outcome) with assistance from a specialist librarian and based on the review aim. Four key databases (Medline, CINAHL, Embase and ProQuest) were searched from inception until the 9th of December 2022, using the Boolean combination AND and OR for “moulage” and “simulation-based training” with relevant MeSH terms and adaptations to suit each database (supplementary material 1). No date or language limitations were applied; however, the ProQuest Central database was restricted to peer-review journals only. A further snowball search of included article reference lists and hand-searching of key simulation journals (Advances in Simulation, Simulation in Healthcare, Clinical Simulation in Nursing INACSL, The British Medical Journal: simulation, International Journal of Healthcare Simulation) was conducted. The systematic review was registered with the International Prospective Register of Systematic Review (PROSPERO) (CRD42021292052). Ethical approval was not required.

### Inclusion and exclusion criteria

Studies were eligible for inclusion if they (1) reported on adult learners (18+ years), including students or workers across all professions; (2) utilised moulage in a simulation-based education or training experience; (3) reported on at least one primary outcome of interest (experience, engagement, satisfaction, preference, preparedness or confidence) with or without a secondary outcome (clinical competency or performance); and (4) employed an empirical research design. Studies were excluded if they reported only on secondary outcomes; were not peer reviewed or were single case studies, editorials, commentary articles or reported only as conference abstracts.

### Study selection and quality appraisal

Search results were exported into a single EndNote 20 library and deduplicated. The results were then uploaded to Covidence© software for title and abstract screening against the inclusion criteria, completed independently by two reviewers (GZ, SD). Papers not excluded during title and abstract screening were retrieved for further independent full-text screening by two reviewers (GZ, SD). Conflicts were resolved either through consensus or discussion with other reviewers (JSP, DR). Included studies were critically appraised independently by two reviewers (GZ, SD) using the Mixed-Methods Appraisal (MMAT) tool [25], with disagreements resolved by consensus or third reviewer (DR). The MMAT is a tool for critically appraising qualitative and quantitative methodology studies using a single tool.

### Data extraction

A data extraction tool was developed to capture key characteristics of included studies. Data extracted for each study included participant type and number, study design, intervention and reported pedagogical theory, comparator, and reported findings. Qualitative studies were tabulated by method of data collection, analysis, identified themes, and supporting quotes. Data extraction was conducted independently by one reviewer (GZ) with all studies checked for accuracy by a second reviewer (SD).

## Results

### Study selection

**Fig. 1:**
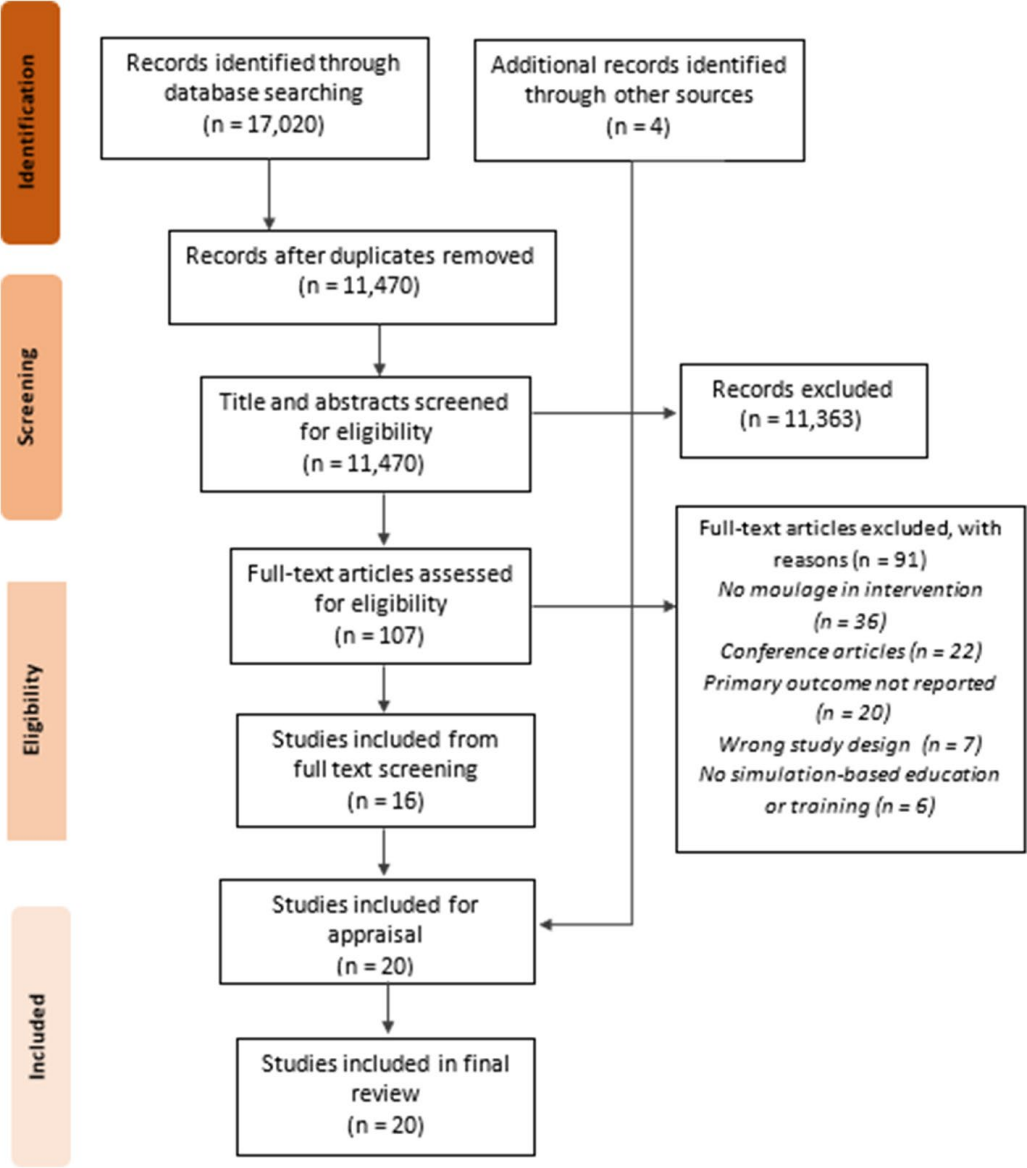
PRISMA Flowchart

Seventeen thousand twenty articles were identified in the database search. Following deduplication, 11,470 articles were screened based on title and abstract. Of these, 111 articles were retrieved for full text review resulting in 16 included studies from the database search (Fig. 1). An additional four studies were identified through snowball and hand searching, resulting in a total of 20 final included studies for quality appraisal. The most common reason for exclusion was not describing or utilising ‘moulage’ within their intervention. Of the included papers within the study, three were duplicated from the previously cited review [24]. The remaining 17 were either studies that were conducted after its review date or not included in the review based on their search strategy and inclusion criteria.

### Quality assessment

The 20 included studies were assessed as variable in quality based on MMAT, against relevant questions according to study design. Nine publications were assessed against the ‘Mixed-Methods’ questions (Domain 5.0); three were assessed against the ‘Quantitative Randomised Controlled Trials’ questions (Domain 2.0) and the remaining eight were assessed against the ‘Quantitative Descriptive’ questions (Domain 4.0). For randomised controlled trials, there was unclear or no blinding of allocation for all studies, and the criterion on appropriate randomisation was rated as no or unclear for 2 of the 3 studies. For quantitative descriptive studies, the most common area that was rated as unclear was the criterion for sampling strategy used (*n* = 3 out of 8 studies), with one of those studies also being rated unclear for the criterion on non-response bias [26]. Mixed methods studies were most frequently rated down for failing to address inconsistencies between quantitative and qualitative findings (*n* = 5 out of 9 studies).

### Study characteristics

The full characteristics of included studies can be seen in Table 1. Most studies were from medical disciplines (*n* = 9), were with undergraduate learners (*n* = 15) and situated at a university setting (*n* = 15). Moulage was utilized to simulate a variety of scenarios. Table 2 outlines the use of moulage in each study and the mediums used to create it.

**Table 1:** Characteristics of studies included in systematic review of moulage interventions *n* = 20

Citation	Study design	Setting/Country	Participant type (number)	Intervention	Comparison	Quantitative findings		Learning theory/framework
						Primary outcomes	*p*-value	
**Flores & Hess, 2018**	Randomised controlled trial	University, USA	Undergraduate, pharmacy students (*n* = 70)	Simulation, simulated participant, moulage (skin disorder)	Paper-based, patient scenario	**Satisfaction:** 5-Point Likert scale post-test, intervention group more satisfied vs. comparator (group 2 only) **Confidence:** 5-point Likert scale post-test, intervention group more confident vs. comparator (group 2 only)	*p* = 0.001^a^ *p* = 0.03^a^ *	None identified
**Garg et al., 2010**	Randomised controlled trial	University, USA	Undergraduate, medical students (*n* = 90)	Simulated participant, teaching session, moulage (skin lesions)	Lecture based teaching session, images	**Satisfaction:** 5-Point Likert scale post-test, 96% of intervention group agreed/strongly agreed session more enjoyable than classroom lectures. 94% agreed more effective teaching method than images.	Not reported *	None identified
**Mills et al., 2018**	Randomised controlled trial, mixed-methods	University, Australia	Undergraduate, paramedic students (*n* = 50)	Simulated participant simulation, moulage (trauma injury)	Wound’s location depicted with sticker	**Immersion:** *Eye-tracking:* Intervention group had higher fixation rate on primary wound. *NASA-TLX:* Higher ‘temporal demand score’ in intervention vs. comparator group.	*p* = 0.004^a^ *p* = 0.04^b^	None identified
**Mills et al., 2020**	Post-test, convenience sample, 2-group crossover, mixed-methods	University, Australia	Undergraduate, paramedic students (*n* = 29)	VR simulation, simulated participant in moulage (mass casualty)	Live simulation, simulated participant in moulage (mass casualty)	**Satisfaction:** *Simulation Design Scale:* Post-test, no significant difference between groups. **Immersion:** *Heart Rate*: avg. HR significantly higher during live vs. VR simulation. *NASA-TLX:* Significantly higher perceived workload in live vs. VR simulation.	*p* > 0.05^c^ *p* < 0.001^c^ *p* < 0.001^c^	None identified
**Pywell et al., 2016**	Post-test, convenience sample, single group	Training facility, UK	Undergraduate, medical students (*n* = 20)	Simulated participant simulation, professional moulage (trauma injuries)	Simulated participant simulation, novice moulage (trauma injury)	**Experience:** 5-Point Likert scale post-test, intervention group had higher face (1) and content (2) validity scores vs. comparator. Not statistically significant.	1. *p* = 0.11^d^ 2. *p* = 0.06^d^	None identified
**Sezgunsay & Basak, 2020**	Pre/post-test, convenience sample, quasi-experimental, mixed methods	University, Turkey	Undergraduate, nursing students (*n* = 73)	Simulated participant, simulation, moulage (pressure injury)	Simulated participant simulation, pictures of pressure injury applied	**Satisfaction:** *Student Satisfaction and Self-Confidence Scale.* No significant difference between groups. **Confidence:** *Student Satisfaction and Self-Confidence Scale.* No significant difference between groups. **Experience:** *Simulation Design Scale*: significantly higher mean score for ‘best design elements’ in intervention vs. comparator.	*p* = 0.30^c^ *p* = 0.10^c^ *p* = 0.04^c^ *	Kirkpatrick’s four-level approach*** (Kirkpatrick & Kirkpatrick, 2005)
**Stokes-Parish et al., 2020**	Post-test, randomised controlled multi-arm, mixed-methods trial	University, Australia	Undergraduate, medical students (*n* = 33)	Manikin simulation, 1. High authenticity moulage, 2. Low authenticity moulage (trauma injury)	Manikin simulation, no moulage, verbal cues given (trauma injury)	**Experience:** 5-point Likert scale, post-test survey. Presence of moulage in both intervention groups positively affected training experience. **Engagement:** 5-Point Likert scale post-test survey. Moulage inclusion/exclusion rated as important and contributed to engagement in all groups. **Immersion:** 5-Point Likert scale, post-test survey. No significant difference between either intervention group or intervention vs. comparators. High authenticity intervention group had least amount of variability in scores.	*p* = 0.03^e^ *p* = 0.02^e^ Not reported.	None identified
**Uzelli & Sari, 2021**	Pre/post-test, randomized, quasi-experimental	University, Turkey	Undergraduate, nursing students (*n* = 62)	Simulated participant, simulation, moulage (IV catheter site)	Manikin simulation, without moulage	**Satisfaction:** *Student Satisfaction and Self-confidence Scale.* Significantly higher overall score in intervention group vs. comparator. **Confidence:** *Student Satisfaction and Self-confidence Scale.* Significantly higher overall score in intervention vs. comparator. **Experience:** *Simulation Design Scale*. No significant difference in scores between groups.	*p* = 0.001^f^ *p* = 0.001^f^ *p* = 0.164^f^*	None identified
**Uzelli Yilmaz et al, 2021**	Post-test, quasi-experimental	University, Turkey	Undergraduate, nursing students (*n* = 66)	Simulated participant, moulage (pressure area injury)	No comparator	**Satisfaction:** 5-point likert scale post-test. Overall satisfaction score = 4.5/5	Not reported	None identified
**DAasta et al., 2019**	Post-test, convenience sample, mixed-methods	Training facility, Italy	Healthcare professionals (nurses, physicians, medical/nursing students) (*n* = 101)	Manikin simulation, moulage (burn injuries)	No comparator	**Satisfaction:** 5-Point Likert scale post-test. Overall satisfaction score = 4.8/5	Not reported.	None identified
**Garvey et al., 2016**	Post-test, convenience sample, single group	Training facility, USA	Post-registration nurses (*n* = 55)	Simulated participant, simulations, moulage (trauma injuries)	No comparator	**Confidence:** 5-Point Likert scale pre/post-test. Positive change noted from pre to post in each question.	Not reported.	None identified
**Hogg & Miller, 2016**	Post-test, purposive sample, single group	University, Scotland	Undergraduate, medical students (*n* = 138)	Simulated participant, simulations, moulage (illness)	No comparator	**Satisfaction:** 5-Point Likert Scale post-test. Overall mean score > 4/5 for all participants. **Confidence:** 5-Point Likert Scale pre/during/post-test. Significant increase in confidence across the three time periods.	Not reported. *p* < 0.001^f^	None identified
**Lazzarini et al., 2011**	Pre/post-test, convenience sample, single group	Training facility, Australia	Post registration, podiatrists (*n* = 16)	Simulated participant, moulage (foot ulcers)	No comparator	**Satisfaction:** 5-Point Likert scale survey post-test. 13/14 participants rated course to be ‘excellent’. 1/14 rated ‘very good’. **Confidence:** Pre/post-test survey. 42% improvement in clinical confidence from pre to post-test.	Not reported.*p* < 0.05^d^	Kirkpatrick’s four levels of evaluation^***^ (Kirkpatrick & Kirkpatrick, 2005)
**Mazzo et al., 2018**	Post-test, convenience sample, single group	University, Brazil	Undergraduate, nursing students (*n* = 100)	Simulated participant, simulation, moulage (pressure injury)	No comparator	**Satisfaction:** *Satisfaction Scale with Simulated Clinical Experience.* 5-Point Likert scale post-test. Overall high scores reported > 89%. **Confidence:** *Student Satisfaction and Self-confidence in Learning Scale.* Overall high scores reported > 89%.	Not reported. Not reported.	Three domains of Bloom’s taxonomy: cognitive, affective, psychomotor (Krathwohl & Bloom, 1956)
**Mcavoy & Kelly, 2020**	Post-test, convenience sample, single group	University, UK	undergraduate, medical students (*n* = 118)	Manikin simulation, moulage (head injury)	No comparator	**Confidence:** 5-Point Likert Scale pre/post-test survey. Average score increase for each item = + 1.3 points. 94.6% participants had overall increased score from pre to post.	Not reported.	None identified
**Saideen et al., 2013**	Post-test, convenience sample, single group, mixed-methods	University, UK	Post registration, emergency medicine professionals (*n* = 12)	Simulated participant, simulation, moulage (burn injuries)	No comparator	**Experience:** 5-Point Likert scale survey post-test. Mean face validity score = 4.1/5 Mean content validity = 4.5/5	Not reported.	None identified
**Santomauro et al., 2020**	Post-test, convenience sample, single group, mixed-methods	Training facility, Australia	Post registration healthcare professionals (*n* = 75)	Manikin simulation, moulage (gunshot wound)	No comparator	**Experience:** 5-Point Likert scale survey post-test. 65.3% strongly agreed manikin’s appearance contributed to learners’ experience. **Immersion:** 5-Point Likert scale survey post-test. 81.3% strongly agreed the moulage enhanced the immersiveness of simulation.	Not reported. Not reported.	None identified
**Shiner, 2019**	Pre/post-test, quasi-experimental, mixed-methods	University, United Kingdom	Undergraduate, radiography student (*n* = 9)	Simulated participant, simulation, moulage (open wound)	No comparator	**Experience:** 5-Point Likert scale, pre/post-test survey. Improvement in all aspects of experience from pre to post except for ‘distraction following seeing open wound.’	Not reported.	PEARLS Framework for Debriefing: Promoting Excellence and Reflective Learning in Simulation (Eppich & Cheng, 2015)
**Shiner & Howard, 2019**	Pre/post-test, purposive sample, single group	University, Scotland	Undergraduate, diagnostic radiography students (*n* = 28)	Simulated participant simulation, moulage (burns injuries)	No comparator	**Preparedness:** 5-Point Likert scale survey pre/post-test. Median shift for one question relating to preparedness.	Not reported.	Empiricism^**^ (Psillos et al., 2020)
**Zorn et al., 2018**	Post-test, convenience sample, single group	University, USA	Preregistration, physician assistant students (*n* = 48)	Simulated participant simulation, moulage (melanoma)	No comparator	**Satisfaction:** 5-Point Likert scale survey post-test. 48% disagreed or strongly disagreed that photographs are superior to moulage in simulation experience. **Experience:** 5-point Likert scale survey post-test. 66% agreed or strongly agreed moulage increased realism of experience.	Not reported. Not reported.	None identified

^a^ Mann Whitney U Test statistical significance set at *p* < 0.05

^b^ One-way multivariate analysis significance set at α = 0.05

^c^ Paired sample *t-*test significance set at *p* ≤ 0.05

^d^ Linear fixed-effects model

^e^ One-way ANOVA statistical significance set at *p* = 0.05

^f^ independent *t*-test

^*^No between post-group results were reported

^**^No a priori theory used but Empiricism used to interpret

^***^Used to evaluate the effectiveness of the intervention

**Table 2:** Summary of moulage interventions in *n* = 20 studies

Citation	Depicted scenario	Simulated Participant	Manikin	Silicone moulds	Special effects make-up	Temporary tattoo	Attachable moulds
Flores & Hess, 2018	Contact dermatitis and drug-induced skin disorder.	**✓**			**✓**		
Garg et al., 2010	Lesions: actinic keratosis, angioma, melanoma, squamous cell carcinoma, and eruptions: contact dermatitis, folliculitis, herpes zoster, psoriasis and tinea corporis.	**✓**		**✓**			
Mills et al., 2018	Trauma injury: pale visage, blood leaking from abdominal wound and spurting from upper thigh wound.	**✓**			**✓**		
Mills et al., 2020	Trauma injury: gunshot wounds, brain injury, blunt force to the head and abdomen, impaled armpit wound, and open fracture to arm.	**✓**			**✓**		
Pywell et al., 2016	Trauma injury: fisherman caught in explosion, drug dealer sustains a flame injury from his lab, pregnant lady suffers scald burn from a hot kettle, and golfer stuck by lightning.	**✓**			**✓**		
Sezgunsay & Basak, 2020	Laboratory setting: Pressure injury level 1–3.		**✓**		**✓**		
	Clinical setting: Pressure injury level 1–3.	**✓**					
Stokes-Parish et al., 2020	Trauma injury: mountain bike accident.		**✓**		**✓**		
Uzelli & Sari, 2021	IV catheter insertion complications: second-degree infiltration and third-degree phlebitis.	**✓**			**✓**		
Uzelli Yilmaz et al., 2021	Pressure injuries on simulated participants on wrists, heel, and tibial region.	**✓**			**✓**		
D’Asta et al., 2019	Chemical, electrical and paediatric burn with inhalation injury.		**✓**		**✓**		
Garvey et al., 2016	Trauma injury: gunshot wounds, bleeding lacerations and bruising/abrasions.		**✓**		**✓**		
Hogg & Miller, 2016	Illness: pale and tired.	**✓**			**✓**		
Lazzarini et al., 2011	Foot ulcer or lesion.	**✓**				**✓**	
Mazzo et al., 2018	Pressure injury stage 3, presence of excaudate, odour and pale skin colour.	**✓**			**✓**		**✓**
McAvoy & Kelly, 2020	Trauma injury: occipital concussion, swelling and bruising to the proximal femur and fingertip bruising to the shoulder.		**✓**		**✓**		
Saideen et al., 2013	15% flame burns on legs caused by clothing catching on fire.	**✓**			**✓**		
Santomauro et al., 2020	Facial gunshot wound created with charcoal dust and base layer of Black fuse FX silicon paint to simulate powder burns.	**✓**			**✓**		
Shiner, 2019	Compound lateral malleolar fracture open wound.	**✓**			**✓**		**✓**
Shiner & Howard 2019	Second- and third-degree burns applied to cheeks, hand and arm.	**✓**			**✓**		
Zorn et al., 2018	Multiple atypical lesion consistent with malignant melanoma attached to dorsolateral wrist.	**✓**		**✓**			

### Impact of moulage on learner experience

For this review, learner experience was captured across several outcome categories including, satisfaction, confidence, immersion, engagement, preparedness, and overall experience. Qualitative results from the nine mixed-methods studies provide further details about how moulage impacted learner experience (Table 3).

**Table 3:** Summary of qualitative findings from *n* = 9 studies reporting the impact of moulage on student experience

Citation	Method of collection	Method of analysis	Identified themes	Quotes
DAasta et al., 2019	Survey questions: • strengths • potential improvements • other comments	Not reported	• Course’s ability to emphasize and teach principles • Opportunity to practice teamwork	*“Make-up and actors felt very real.”* *“*In situ *simulation makes me better immerse into the scenario.”*
Sezgunsay & Basak, 2020	Semi-structured feedback form with questions: • How did you feel? • Did you face difficulty? • How did the lab practice affect, your pressure injury assessment in the clinical setting?	Content analysis, manifest style: • Decontextualization • Recontextualization • Categorisation • Compilation	**Difficulties:** • Intervention group was unfamiliar with what they were seeing • Control group found it hard to differentiate between tissue, bone, tendon visuals. **Positive feedback:** • Simulation supported the integration of theory and practice. • Intervention group found the simulation more realistic **Negative feedback**	*“…the staging was easy for me; it was good for me to work on the moulage beforehand.”* *“Touching and communicating with a real patient is different, of course, but I felt less stressed in the simulation.”* *“Pre-defining on the moulage made it visually a lot easier and more memorable.”*
Mills et al., 2018	Semi-structured face-to-face interview immediately post-simulation.	Pragmatic, action-research oriented, interpretive inquiry approach. Audio-recorded, transcribed, QST NVivo software.	**Visual cues:** • Highly realistic • Blood served as visual cue to provide immediate feedback to guide decisions • Gave indication of severity of condition **Realism:** • Moulage would have facilitated them taking the task more seriously **Immersion:** • Moulage helped them treat the patient faster	*“I was able to walk-in and just immediately see what the problem was.”* *“You don’t feel that urgency for it, or the same urgency as you would if you actually saw that”* *“The process is faster…you’re getting that instant feedback.”* *“Staying calm under pressure and keeping a clear head and that sort of thing. The more sort of crazy factors like that you throw in, the better equipped we’re going to be by the time we graduate.”*
Saideen et al., 2013	In-depth interviews and focus groups completed post-simulation	Audio-recorded, transcribed verbatim and coded based on topic guide.	• Participants’ authentic behaviour • Increased cognitive load • Increased confidence • Applicability of the scenario as a training tool	*“Everything. The sounds…the child, her screaming, make-up feedback.”* *“I will still be thinking about this scenario…previously thought to be a ‘daunting task’ but in fact it is ‘manageable’”*
Stokes-Parish et al., 2020	Individual interviews post simulation, using video-stimulate recall technique.	Audio-recorded, transcribed verbatim. Grounded Theory technique: • familiarisation • initial code • categorical coding • making meaning	**Rules of simulation:** • Aware they were in a simulation yet mentally processing the conditions of simulation vs. reality. **Believability:** • Background awareness of simulation depending on authenticity presented. **Consistency of presentation:** • Consistency of presentation of visual cues. **Personal knowledge:** • Previous simulation experiences leading to treat future simulations with less believability	“*As soon as I looked and then saw it was like crystal clean…it just like kind of pulls you back in, okay it’s a simulation.”* *“[I]…have to switch out of the scenario to check things out. In real life you can either see it’s happening or it’s not.”* *“The moulage is good and it’s showing what it’s meant to…but if it’s just like a sticker or something that says, ‘blood here’, then that might detract from the situation”*
Mills et al., 2020	Focus groups with eight participants in each, post-simulations	Audio-recorded, transcribed verbatim, QST NVivo software. Pragmatic, action-research orientated, interpretive inquiry approach.	• Virtual Reality (VR) experience graphically realistic to the simulation • VR unable to replicate the human interaction and emotional immersion • VR experience a ‘steppingstone’ to live simulation	*“I found the VR really good just to practice that skill alone without the extra stress”* *“I think the live one was a bit more intense, like more full-on.”*
Shiner., 2019	Semi-structured focus groups post first clinical placement.	Data transcribed verbatim. Phenomenological analysis six-step approach. Data reviewed blindly in duplicate and themes agreed upon together.	**Engagement with wound:** • Distracted by curiosity • Intervention group had a more pragmatic response to seeing their first wound compared to control **Emotional engagement:** • Challenged by the unknown • Early career experiences **Developing professional self:** • Continual challenges • Processing past experiences • Coping mechanisms **Simulation impact:** • Positive experience to prepare for seeing open wounds **Building relationships**	*“Because it hasn’t phased me, I’m not nervous or anxious about it.”* *“You can’t go back from that, you can’t unsee it”* *“Bloods red, reds a colour that’s it.”* *“Treating it as a patient and not a body part.”* *“Oh no it prepared, fully. For me it did because it made me think outside the box.”*
Shiner & Howard., 2019	Focus groups conducted three months post experience	Audio-recorded, transcribed verbatim. Thematic analysis using six-phase coding process. Concordance check between two researchers.	**Patient centredness:** • What others see • Non-verbal cues/reactions **Learning:** • Experiential learning had improved their memory recall for their exam **Realism:** • Wounds appeared realistic • Thought it was an actual burns patient.	*“I saw some people’s faces and they looked pretty scared.”* *“During the OSCE I thought back and its kinda helped.”* *“The makeup was really realistic looking I couldn’t tell it was makeup at first.”* *“To be honest, you couldn’t really hide it was intense.”* *“For me it felt like definitely one of the topics I was most prepared for as I’m a visual learner.”*
Santomauro., 2020	Anecdotal comments from the simulation staff.	N/A	• Participants are taken aback when they first see the manikin. • Participants display caution and apprehension when examining.	N/A

### Satisfaction

A total of 11 studies reported on participant satisfaction (Table 1). Most studies (*n* = 6) utilised post-survey design and found higher learners’ satisfaction with the use of moulage. Of the other five studies that utilised moulage during simulation and compared the findings to a comparator scenario, three in total reported statistically significant higher satisfaction with the moulage intervention versus the comparator.

### Confidence

A total of 8 studies reported on participants’ improvement in confidence regarding clinical management of the relevant medical presentation, however only three [27–29] were statistically significant.

### Immersion and engagement

Four studies in total reported on participants’ level of immersion and engagement with the simulation experience. In two studies, participants highly rated the inclusion of moulage as an important and strong contributor to the level of immersion or engagement they felt with the experience [30, 31].

### Preparedness and experience

A total of nine studies reported on the level of preparedness for practice and overall experience as part of their quantitative findings. Overall, moulage contributed positively to learners’ experience by improving self-reported preparedness for practice. One study [32] reported that although moulage improved participant experience, feelings of distraction increased after seeing an open wound.

### Impact of moulage on knowledge and clinical performance

#### Knowledge

Five studies reported on level of knowledge using a pre-post-test assessment design. Results varied between studies, however the majority saw improvement in knowledge scores.

#### Performance

Of the six studies that reported on the performance and clinical competency of participants, three studies [10, 29, 33] found the intervention group to have higher mean scores compared to the control group.. Two studies measured time-to-action, however, had mixed results [21, 31].

### Pedagogical theories informing moulage practice

Five studies mentioned a pedagogical framework or learning theory. Two studies used Kirkpatrick’s four-level approach in the evaluation of their interventions [34]. The remaining three mentioned one of the following theories or frameworks; Bloom’s Taxonomy Educational Learning [35]; Promoting Excellence and Reflective Learning in Simulation [36]; and Empiricism [37]. One study [38] referenced the pedagogical theory as underpinning their intervention, one utilised it for guiding the reflection phase of the simulation [32] and the remaining three referenced a theory either to interpret [39] or evaluate their results [33, 40].

## Discussion

This systematic review explored the effects of moulage in SBE on learner experiences and described the pedagogical theories underpinning the simulation experience. The results suggest that the use of moulage does impact learner experience, by improving learner satisfaction, confidence, and immersion within the task. However, moulage did not improve knowledge attainment and performance, although overall evidence was weak. The use of moulage within included studies was limited to a small range of health professions (predominantly medical and nursing disciplines). Only a handful of papers (*n* = 5) identified pedagogical theories as informing the research design.

Our findings of improved participant satisfaction and confidence regarding clinical skills, supports simulation as an effective learning technique to increase self-efficacy. Self-efficacy is an individual’s perception of their ability to achieve a goal, and while it is not a reflection of their actual capabilities, it can affect performance and achievement [27, 41, 42].

Moulage contributes to a realistic simulation experience and consequently, improves learner engagement [24]. Engagement in simulation has been described as “the state in which the participant is observed to be actively interacting with the simulation as if it were real” [31] [31] Our review supports this, as studies consistently reported that moulage contributed to the perceived engagement of participants A proposed reason for this is that moulage provides visual cues for learners to guide their actions, without disrupting the flow of the experience [21, 31]. Participants have previously noted that the benefit of the moulage was that they did not need to “switch out” of simulation mode to gather cues from other sources [31].

A similar, but separate concept of engagement is immersion which is the “subjective impression that one is participating in a comprehensive, realistic experience” [43]. An interesting simulation modality that emerged from our review, was virtual and augmented realities [44]. In the included study, simulated participants with applied moulage, were filmed in 360-degree, virtual reality compatible footage to simulate a live mass-causality scenario [44]. Paramedic students wore a virtual reality headset that immersed them in the footage and allowed them to gather basic clinical information and allocate a triage position. While no difference in satisfaction between the live or virtual reality simulation experience was found, participants noted the lack of human interaction and emotional immersion as a limitation of virtual reality technology. However, virtual reality may be a viable option for improving immersion given its minimal maintenance costs and ability to expose learners to situations difficult to replicate with traditional techniques, such as those that are dangerous or rare (Mills et al., 2020). Augmented reality (whereby virtual objects appear to coexist in the same space as the real world), may present an interesting approach for moulage to include human interaction whilst retaining the desirable traits of virtual reality simulations, such as immersion and cost-effectiveness.

Simulations have been found to improve empathy and communication skills in healthcare learners [45, 46]. Empathetic encounters with healthcare professionals have been shown positive results for patient care [47], whilst, conversely, a lack of empathy can result in increased risk of harm to patients [48, 49]. Our findings suggest moulage may aid in developing participants’ empathy by preparing them for potentially distressing scenarios with simulated wounds and illnesses [32, 39]. One reason for this may be its ability to develop learned psychological responses to uncomfortable imagery without causing them to lose comprehension of the patient’s feelings and emotions [50], which is what occurred in the Shiner & Howard (2019) study. The simulation with moulage prepared them emotionally and assisted in maintaining person-centredness [39]. Notably, none of the included studies did incorporated smell, which may be a topic for consideration when it comes to preparedness. There is some contention on whether smell can be considered a component of moulage, however, experts could not agree that smell was moulage in a 2017 consensus study [14]. Despite this, smell could create an additional stimulus for experience or emotion. SBE with moulage may assist learners to better display empathy to patients and in situations reflecting more confronting cases prior to practice, as well as emotionally preparing them in a psychologically safe environment.

There was limited used of pedagogical theory in the development of moulage interventions, reported by only one of our included studies [38]. However another four described a pedagogical theory in the manuscripts, or provided other evidence of considering learning approaches such as Bloom’s Taxonomy [35]. When a theory was utilised to evaluate the simulation-based experience, student performance was greater despite no difference in knowledge to a control group [33]. Previous researchers have suggested that the use of a learning theory within simulation-based education and training provides a more structured experience that integrates effective learning attributes and skills [51]. Greater use of learning theories in the development of moulage interventions may improve outcomes for learners beyond experience.

Several theories could inform moulage practice in simulation, as described earlier. Relevant theories include realism and authentic learning [11, 52]. Another theory that might have application is the theory of visual attention, whereby the eyes constantly scan the visual field to determine areas of priority [53]. This may have relevance when it comes to moulage, due to its focus on visual cues. For example, in the virtual reality study described earlier (Mills et al. (2018), the theory of visual attention would have been to the use of eye tracking methodology to determine paramedicine student engagement with moulage [21]. Similarly, authentic learning theory could have been applied to Flores & Hess’ (2018) work, in which they utilised moulage to improve pharmacy students’ ability to assess skin disorders instead of pictures [27].

We found that moulage in SBE is rarely utilised with participants from allied health professions, with only one included-study evaluating the use of moulage to improve podiatrists’ confidence in foot ulcer management [40]. Notably, we did find published work in non-health fields, such as emergency services and military fields, however these studies did not meet our inclusion criteria [20, 54, 55]. For this reason, the use of moulage may be broader than what is reported in the literature. The lack of use in allied health may be attributable to the costs associated with high quality moulage techniques and the constraints of time. However, given the findings of this review which suggest that communication skills may be impeded in confronting situations and the importance of communication and emotional intelligence for allied health professions, such as dietetics [5], there is cause for more robust research in this area.

Considering the direction for future research, it may be useful to steer focus away from objective outcomes such as knowledge or clinical skill performance, as our findings suggest that moulage has minimal impact on these. Instead, we recommend to further explore: 1) what contributes to a beneficial learning experience using qualitative enquiry methods, 2) the role of emotional preparedness and moulage, 3) moulage and the return on investment, 4) the role of moulage in empathy development, 5) the barriers to disciplines embedding moulage in practice, and 6) the impact the level of the learner has on moulage importance in simulation.

The strengths of this review include the comprehensive search strategy across both databases and relevant journals, with no restriction on profession, language or date, thus maximising the studies captured. Independent researcher screening, data extraction and appraisal of studies provided confidence in the robustness of the methods. Inclusion of a broad range of primary outcomes and study designs captured a breadth of learners’ experiences relating to the use of moulage. However, there are limitations that need to be considered. Firstly, there were large discrepancies in quality of study design; notably the high number of included studies following a single-site, one-test point design. 12 of the 20 studies had no control group or comparator arm, resulting in difficulties measuring the effectiveness of the outcome change. In addition, four of the 20 studies did not provide sufficient justification of which statistical tests were used to analyse the data, thus reducing the confidence in the results provided. Although three studies utilised a validated tool to evaluate study outcomes, the data was presented using different statistical analysis, which limits comparisons between studies. Additionally, most of the studies utilised self-reported surveys to collect data, which is not a valid method to evaluate performance.

## Conclusion

The use of moulage within SBE and training can play an important role in the experience of the learner. Moulage contributes to improved learner satisfaction, immersion, confidence, and may contribute to empathy development, but not necessarily knowledge improvement. The opportunities for future research are immense, spanning pragmatic considerations and pedagogical enquiries. There is a continued need for higher quality evidence with robust study designs that are based on pedagogical theories and learning frameworks, evaluating the use of moulage against designated comparator interventions or controls. We recommend that researchers resist the temptation to focus on knowledge and focus on emerging areas of moulage, such as empathy and emotional preparedness.

### Supplementary Information

**Additional file 1.**

## Data Availability

All data generated or analysed during this study are included in this published article [and its supplementary information files].
