# Supplementary material for: The impact of moulage on learners’ experience in simulation-based education and training: systematic review
Source: BMC Med Educ. 2024 Jan 3;24:6. doi: 10.1186/s12909-023-04976-w (PMC10765801; doi:10.1186/s12909-023-04976-w)
Supplement: Supplementary file 1 — Additional file 1. [file 12909_2023_4976_MOESM1_ESM.docx]

Supplementary Material

# Supplementary material 1. Database search strategies

| Database | Search Strategy | Results | Date |
| --- | --- | --- | --- |
| PubMed | ((Simulation training[MeSH] OR Simulation training[tiab] OR Practical task[tiab] OR Clinical task[tiab] OR Practice scenario[tiab] OR Assessment[tiab] OR Human patient simulations[tiab] OR "Education, Medical/methods"[MAJR])) AND ((Moulage[tiab] OR Fidelity[tiab] OR Tattoos[tiab] OR "Anatomic models"[tiab] OR "audio-visual aids"[tiab] OR "medical illustration"[tiab])) | 4688 | 09.12.22 |
| CINAHL | ((TI Moulage OR AB Moulage) OR (MH Moulage+) OR (TI Fidelity OR AB Fidelity) OR (TI Tattoos OR AB Tattoos) OR (TI "Anatomic models" OR AB "Anatomic models") OR (TI "audio-visual aids" OR AB "audio-visual aids") OR (TI "medical illustration" OR AB "medical illustration")) AND ((MH "Simulation training"+) OR (TI "Simulation training" OR AB "Simulation training") OR (TI "Practical task" OR AB "Practical task") OR (TI "Clinical task" OR AB "Clinical task") OR (TI "Practice scenario" OR AB "Practice scenario") OR (TI Assessment OR AB Assessment) OR (TI "Human patient simulations" OR AB "Human patient simulations") OR (MM "Education, Medical/methods"+)) | 4711 | 09.12.22 |
| EmBase | (Moulage:ti,ab OR Moulage/exp OR Fidelity:ti,ab OR Tattoos:ti,ab OR "Anatomic models":ti,ab OR "audio-visual aids":ti,ab OR "medical illustration":ti,ab) AND ("Simulation training"/exp OR "Simulation training":ti,ab OR "Practical task":ti,ab OR "Clinical task":ti,ab OR "Practice scenario":ti,ab OR Assessment:ti,ab OR "Human patient simulations":ti,ab OR "Education, Medical/methods"/exp/mj) | 5698 | 09.12.22 |
| Proquest Central | (TI,AB(Moulage) OR TI,AB(Fidelity) OR TI,AB(Tattoos) OR TI,AB("Anatomic models")) AND (TI,AB(“Simulation training”) OR TI,AB(Assessment) OR MJMESH.EXACT("Education, Medical/methods")) | 3010 (Peer Reviewed) | 09.12.22 |
